# Australians' experiences of COVID-19 during the early months of the crisis: A qualitative interview study

**DOI:** 10.3389/fpubh.2023.1092322

**Published:** 2023-02-23

**Authors:** Deborah Lupton, Sophie Lewis

**Affiliations:** ^1^Vitalities Lab, Centre for Social Research in Health, Social Policy Research Centre, UNSW Sydney, Kensington, NSW, Australia; ^2^School of Health Sciences, Faculty of Medicine and Health, University of Sydney, Camperdown, NSW, Australia

**Keywords:** COVID-19, Australia, qualitative, interviews, socio-spatial analyses, crisis, life experiences, sociology

## Abstract

**Introduction:**

The COVID-19 crisis has wrought major changes to people's lives across the globe since the beginning of the outbreak in early 2020. The "Australians' Experiences of COVID-19” qualitative descriptive study was established to explore how Australians from different geographical areas and social groups experienced the COVID-19 crisis.

**Methods:**

Three sets of semi-structured interviews, each with a diverse group of 40 adults across Australia, were completed between 2020 and 2022. This article reports findings from the first set of interviews, conducted by telephone in mid-2020.

**Results:**

The participants discussed their experiences of living through this period, which was characterized by strong public health measures to contain the spread of COVID, including a national lockdown and border closures. Interview fieldnotes and verbatim transcripts were used to conduct an interpretive thematic analysis. The analysis is structured around the following five themes covering the quotidian and affective aspects of participants' lives in the early months of the COVID crisis: “disruption to routines;” “habituating to preventive measures;” “social isolation and loneliness;” “changes to work and education;” and “little change to life.” A sixth theme concerns how participants responded to our question about what they imagined their lives would be like after the pandemic: “imagining post-COVID life.”

**Discussion:**

The crisis affected participants' experience of daily life variously according to such factors as their social circumstances and obligations as well as their histories of illness, making visible some of the unequal social and economic effects of the pandemic across different genders, ages, localities and socioeconomic groups. Our participants fell into three roughly equal groups: (i) those who found the lockdown and associated restrictions very difficult; (ii) those who reported feeling barely affected by these conditions; and (iii) those who found benefits to the “slowing down” of life during this period.

## 1. Introduction

Since erupting in early 2020, the COVID-19 pandemic has dramatically affected all regions of the world, with new variants and subvariants of SARS-CoV-2 continuing to evolve and causing new outbreaks. Several million lives have already been lost to COVID worldwide. In most countries, everyday lives and the economy as well as people's health have been severely disrupted (1, 2). Throughout the pandemic, many healthcare systems have been strained by caring for unprecedented numbers of seriously ill patients with COVID (3). Social research is urgently needed to document people's everyday experiences of living in this time, how different countries and governments are addressing the pandemic, what measures and policies have been most effective and the consequent social changes.

A multitude of studies have now been conducted globally on people's experiences of COVID, pointing to the often very different conditions and outcomes for people of the crisis depending on aspects such as their nation's COVID prevention policies, its provision of healthcare and welfare support, treatment of socioeconomically disadvantaged people and other vulnerable groups such as older people and people living with pre-existing medical conditions. Socio-spatial dimensions are crucial to these experiences. As Sparke and Anguelov (4) note, there are geographies of infection, vulnerability, blame, immunization, interdependency, care and resilience to identify. These socio-spatialities can be widely varied even within regions (5). Research in countries around the world has drawn attention to the emotions felt during the initial months of the pandemic, as people faced loneliness and feelings of isolation during lockdowns and quarantine periods, as well as fear and anxiety about becoming ill or dying from a previously unknown and still mysterious virus (6–8). The exacerbation of pre-existing socioeconomic disadvantage due to COVID lockdowns and other restrictions as well as inadequate healthcare has been identified across the globe (2, 5, 9, 10). In many countries, young people in particular have been badly affected by lockdowns (11), as have women faced with financial insecurity or attempting to work from home at the same time as juggling caring or educating children unable to attend school or childcare (12). People living in conditions of socioeconomic disadvantage have borne the brunt of the health and economic impacts of the COVID crisis (13).

In the first year of the pandemic, experiences varied quite widely across nations, depending on the types of public health measures that were introduced and the resultant case numbers and death rates. For example, people in nations where strong restrictions were implemented early found life to be very different from those where COVID management was poor or significantly delayed (2). Australia is one of the few nations in which governments and health agencies implemented effective public health measures such as border closures and lockdowns early on, pursuing an elimination strategy that was largely successful during the first year of the pandemic (14–16). The “Australians' Experiences of COVID-19” study was designed to investigate the socio-spatial aspects of everyday life in this wealthy nation. This study is a continuing investigation across several years of the COVID crisis, with three stages completed to date. Stage 1 was conducted when the ancestral SARS-CoV-2 was first spreading around the world, Stage 2 in late 2021, when the second COVID wave led by Delta variant had achieved dominance in infections, both in Australia and worldwide, and Stage 3 in late 2022, following the outbreak of the Omicron variant and the “living with COVID” phase of Australia's approach to management of the pandemic (15, 17).

This article reports findings from the first set of interviews, conducted by telephone in mid-2020 with a diverse group of 40 adults across Australia. Following overviews of the COVID crisis in Australia and the findings of previous research on Australians' attitudes and behaviors in response to the pandemic, we provide further details of our methods and participants. Our analysis focuses on the participants' responses to questions about how their everyday lives had changed during this period, what were the most challenging aspects they faced and what they imagined their lives would be like once the crisis has passed. The presentation of findings is structured around these questions and the topical themes we identified in participants' responses.

## 2. Background: The COVID-19 crisis in Australia

Like most countries, Australia has faced rapid changes in COVID risk and management of that risk since early 2020 (15, 16). The disease that became known as COVID-19 was reported on the last day of 2019 and was declared as a pandemic by the World Health Organization on 11 March 2020 (18). During the first 6 months of the COVID crisis, Australians, together with the rest of the world, were learning about this new coronavirus SARS-CoV-2 and the disease it caused. Australian governments were confronting the problem of how best to manage the pandemic and limit the force of both health and socioeconomic impacts. In January and February 2020, COVID was viewed as a “distant threat” to Australians (17), with the first case (a traveler from Wuhan. China) identified on 25 January (14). The situation rapidly changed in mid-March, however, following the first cases identified of spread of the virus within Australia and the declaration by the World Health Organization that COVID was characterized as a pandemic. Australian governments began to act decisively to implement strong controls to limit the spread of the virus within the nation's borders (15).

Australia is governed by both federal and state government systems. There is a Commonwealth Department of Health and Aged Care, a Chief Medical Officer and a Minister for Health and Aged Care. Additionally, each state or territory has its own government, leader of the government (Premier), health department, health minister and Chief Health Officer. From 23 March 2020, the Australian federal government imposed an unprecedented severe lockdown on the whole nation in the attempt to “flatten the curve” of COVID infections. Schools and workplaces were closed, and many businesses were forced to shut down resulting in high numbers of people losing their jobs. Most people were expected to stay confined to their homes, with the exception of workers such as those in healthcare, supermarkets, delivery services and other essential services. International borders were closed, so that travel to and outside Australia was essentially banned, with exemptions provided only in exceptional circumstances (14, 19).

Despite a conservative federal government being in power at the time, strong social welfare measures (such as the JobSeeker and JobKeeper programs) were implemented to provide financial support to people who were unemployed or had lost income due to mandated business closures (17). State/territory-based leaders also played major roles in COVID management and control, informing publics about restrictions, announcing state-based case and death numbers and imposing local restrictions such as internal border closures and lockdowns (20). By early April 2020, these strong measures had begun to take effect and COVID case numbers were falling. From late April, governments began to loosen restrictions and the lockdown was gradually lifted. Australia moved into the “COVID Zero” phase (17), in which governments sought to eliminate cases of the disease (15, 16).

Most assessments of global responses to COVID agree that Australia's management of COVID in 2020 and 2021 was among the most effective (10, 19). Excess mortality in Australia during this period fell while similar nations endured massive death rates and hospitalizations per capita due to severe COVID (16). However, by the end of 2021, and with the introduction of COVID vaccines, federal and state governments were highly wary of imposing further restrictions. They decided on a “living with COVID” strategy, in which government and public health measures were scaled down significantly and instead there was an emphasis on personal responsibility for managing exposure to COVID (17). Unfortunately, the Omicron variant then reached Australia, and into 2022 COVID cases and deaths rose exponentially (16).

## 3. Previous research on Australians' early experiences of COVID

Several quantitative surveys were conducted in Australia during the early phase of the pandemic, identifying the effects of lockdown on Australians' mental health and feelings of wellbeing. An online survey studying self-reported acute mental health responses during the first COVID wave found that most respondents reported that their mental health had worsened since the outbreak. One quarter of the respondents were very or extremely worried about contracting COVID and half were worried about family members or friends becoming infected (21). Researchers focusing on the state of Western Australia identified that respondents' experiences of the national lockdown compared with the post-lockdown period were characterized by significantly lower levels of physical activity, poorer mental wellbeing and sense of control over one's life, greater feelings of loneliness and higher consumption of unhealthy food, sugary and alcoholic drinks (22). Surveys have also demonstrated similar gendered effects to those found in other countries, with Australian women shouldering most of the burden of caring responsibilities and loss of income during the national lockdown, with little recognition from their employers of this additional unpaid labor (23).

Qualitative studies investigating Australians' experiences of the first COVID wave in greater depth have also identified the stresses on their mental health and quality of life. Research with marginalized or vulnerable groups that were already dealing with significant challenges identified decreased wellbeing. Studies involving gay and bisexual men reported loss of ties during the national lockdown to the communities and leisure, sexual activity and social spaces in which these men found a sense of belonging, accompanied by feelings of loneliness and loss (24). People living with cancer also struggled with new challenges, including loss of access to healthcare and greater feelings of vulnerability, dread and fear (25). Interviews with middle-aged women found that some were turning to alcohol to alleviate the stress of the lockdown and worries about the risks they faced from the pandemic (26).

However, other studies have highlighted the “silver linings” that Australians described during this time, including support for the public health measures due to feelings of safety and security, gratitude that the government was taking strong action to protect its citizens, and the opportunity to make stronger connections in the community, reset life priorities and to build resilience (27). While some people's mental health was negatively affected by lockdowns, others developed effective coping strategies and ways of offering social support and connecting to their communities (28). Further qualitative studies have also shown that many Australians adapted relatively well to staying at home during this phase of the pandemic, embracing video messaging and texting as a way of maintaining intimate connections with friends and family (29) and adapting home spaces for remote working arrangements (30) and exercise (31). Analysis of free-text responses to a questionnaire completed by women with young children living in a rural area of Australia found that they reported facing challenges such as worrying about their family members' wellbeing, their children's health and development, and financial and employment issues. However, these women also discussed ways that the lockdown had made their lives more enjoyable or easier, including more relaxed family routines, greater opportunity to spend quality time with their family members and a positive impact on their children's development (32).

The findings from Phase 1 of the “Australians Experiences of COVID-19” project build on and flesh out some of these quantitative and qualitative findings by providing further in-depth insights into what life was like for people during this period. The research questions of this project were broad, seeking to identify how adults living in Australia from a wide range of geographical locations across the nation and across different life stages and occupations dealt with COVID risks and responded to prevention strategies.

## 4. Materials and methods

### 4.1. Study design

The “Australians' Experiences of COVID-19” project was designed as a qualitative descriptive study, with separate sets of interviews conducted in each of 2020, 2021, and 2022. In Phase 1 a total of 40 indepth semi-structured interviews were conducted by the second author with adults living in Australia between late May and late July 2020. We chose to use telephone calls to conduct the interviews because face-to-face interviews could not take place during this period of physical distancing restrictions. Adopting this method also meant that we could easily involve people living across the vast continent of Australia, including those residing in rural and remote locations who are often excluded from social research, and therefore achieve diversity in our participant group. While telephone interviews do not allow for observations of interviewees' bodily demeanours and other visual cues, they can still generate rich and detailed accounts. Indeed, sometimes the more anonymous nature of the interview encounter can encourage a more expansive discussion, particularly of sensitive topics (33).

### 4.2. Ethical aspects

The study was approved by the UNSW Sydney Human Research Ethics Committee (approval ID HC200292). All participants provided informed consent prior to the interview. As part of this process, in the project information provided to them, participants were told that they could refuse to answer any questions if they felt uncomfortable or distressed and that they could withdraw from the interview at any time. Contact details for counseling services were provided if participants felt that they needed support following the interview. We offered a gift card to thank and compensate participants for their time. To maintain confidentiality when reporting findings from the interviews, participants were assigned a pseudonym and all contextual identifiers were removed from the transcripts. The people who identified as transgender and gender non-conforming are referred to with the pronoun “they.”

### 4.3. Participants and setting

We set sub-quotas in our recruitment to ensure a heterogeneous interviewee group with a spread of participants across gender, age group, and geolocation. Interested potential participants responded to an advertisement about the study on Facebook. At the time this study was carried out, figures on Australian Facebook use show that 60 percent of all Australians were regular Facebook users, with 50 percent of the Australian population logging on at least once a day (34). Using this method of recruitment proved to be fast and effective, and we easily met our sub-quotas. Once enough people had responded in each sub-quota, we stopped recruiting for it and began to fill the other sub-quotas. Table 1 shows participants' sociodemographic characteristics. The age range of participants was from 18 to 76 years. None of the participants had tested positive to COVID at the time of interview, reflecting the relatively low case numbers in Australia during this period in the pandemic and the success of the preventive measures that had been put into place by the federal and state/territory governments.

**Table 1:** Participant sociodemographic characteristics (*n* = 40).

**Gender identification**	
Female	19
Male	18
Other	3
**Age group**	
18–29	10
30–49	9
50–69	13
70+	8
**Location**	
Metropolitan	17
Regional	13
Rural/Remote	10
**Education**	
University	19
No university	21
**Ethnic/racial identification**	
Anglo-Celtic/European	33
Indigenous Australian	1
Asian	3
Central/South American	2
Middle Eastern	1

### 4.4. Data collection

We used a semi-structured interview schedule (see Supplementary material) which allowed participants to elaborate on their answers. Participants were asked to talk about how they had first heard about COVID-19, what the most helpful or useful source of information for them to learn about the coronavirus, how their everyday lives had changed during lockdown, what have been the most difficult or challenging aspects, how they have coped with these difficulties, what services they had used and their view on how well the Australian federal and state governments have dealt with the crisis. The final questions invited the participants to imagine what life would be like once the crisis had passed. All interviews were audio-recorded and professionally transcribed. The second author wrote fieldnotes immediately following each interview, providing initial details of the participants' responses. These field notes were supplemented by the addition of further details and direct quotations by both authors using the interview transcript files once they had been completed by the professional transcribing service.

### 4.5. Analysis

Our analysis of the interviews drew on these field notes together with further reference to the interview transcripts. We used an interpretive inductive thematic approach, which involves identifying patterns across the interview responses rather than seeking to test pre-established hypotheses. This post-positivist approach to social inquiry is directed at identifying “making the mundane, taken-for-granted, everyday world visible” through interpretative and narrative practices (35). As sociologists, we were interested in identifying the logics that people drew on when explaining their experiences, the social relationships, connections and practices in which they took part and how they described their emotional responses (what it felt like to live during this time). This is an “analytically open” approach which attempts to explore the multi-faceted dimensions of everyday life (36). Our approach therefore did not follow a standard “coding protocol,” as we do not view the process as a linear “coding” process. Instead, our analytical process was as follows. Both authors independently began their analyses, identifying themes and cutting and pasting relevant excerpts from the interview transcripts under these themes. The authors then iteratively collaborated in deciding on which themes to highlight and in writing the analysis presented here, passing versions of our analyses back and forth and refining and editing each other's work as we did so. We acknowledge that any researcher comes to analysis with their own perspective and that analytical collaboration is a process of sharing and mulling over other collaborators' interpretations as we reach consensus over how to present our findings. Adopting this reflexive approach means eschewing a positivist perspective on qualitative research in which proof of “rigour,” “objectivity” and “reliability” is sought. Instead, researcher subjectivity is treated as resource for interpretation of research materials that can always only ever be partial and contextually situated (37).

## 5. Results

The analysis is structured around the following five themes that we developed together. Five of these themes cover the quotidian and affective aspects of participants' lives the early months of the COVID crisis: “disruption to routines;” “habituating to preventive measures;” “social isolation and loneliness;” “changes to work and education;” and “little change to life.” A sixth theme concerns how participants responded to our question about what they imagined their lives would be like after the pandemic: “imagining post-COVID life.”

### 5.1. Disruption to routines

Following the implementation of the national lockdown, people were adjusting to the realities of the COVID crisis and the accompanying restrictions and other changes in their lives that the government had implemented to contain the spread of SARS-CoV-2. The participants described many examples of their everyday routines and practices being thrown into disarray due to the COVID crisis, from the loss of employment to significant disruptions to sleep, diet and exercise habits, due to working from home. As James (aged 26), told us:

I'm usually an early riser, I'll get up pretty early, get into work early. Because I'm working from home, I'm not waking up as early and I'm also going to bed a little bit later throughout the day. It's definitely changed my routine.

Several participants reported intense feelings of stress and anxiety during the national lockdown. For some people, like Riley (aged 29), this led to difficulties with sleeping. They noted that the first week of the lockdown “I was a real mess. I was waking up in the middle of the night in a cold sweat.” Others, who found themselves spiraling into depression, began to sleep more. Amala (aged 21) talked about rarely leaving her bedroom and sleeping most of the day because she no longer had “anywhere to be” after losing her casual retail job and being unable to attend university. The loss of the routine and structure of work and university negatively affected her mental health, self-worth, and sense of belonging. Though she recognized that the social conditions surrounding COVID-19 had been imposed on her, she still viewed her struggles as partly a personal failing:

[My] mental health [has been affected] because if you are staying at home the whole time and you just don't feel like get out from your bed... I just became really lazy... some days I had really difficulty to get up from my bed and reach out to my computer and do my stuff.

Others described trying to deal with feelings of uncertainty, stress and the loss of “normal life.” Peter (aged 39) commented that for him, the greatest challenge he faced was coping with these feelings:

It's such a new disease, and people are trying to understand it and what it does. Just that sense of the unknown, and also just the loss of some markers of normal life, weddings, and gatherings, personal touch, just normal markers of life, that kind of thing are probably the hardest part. Obviously, there's the economic impact and the uncertainty around that.

Some people recounted how their eating and exercising habits had changed for the worse. They found that they did not feel safe leaving the home to take their usual outside exercise, such as a walk, and therefore this habit fell away. As Joe (aged 41) put it:

I've put on a lot of weight. Yep. My diet's been probably pretty shocking… I definitely haven't been going out and walking and doing much exercise. I know they said you could, but it's still so–I just felt uncomfortable going out there.

Similarly, Michael (aged 56), who became unemployed during the national lockdown, said that the stress and anxiety of being stuck at home had led to what he called a “downward spiral” of using alcohol as a coping mechanism:

after sitting at home for so long trying to not go anywhere, you just find that, yeah, you just turn to–[it's] lunchtime, I might as well have a beer now and off you go.

In contrast, people who had been able to maintain their employment and were financially secure described working from home as offering benefits such as affording “more time” for exercise. For some, new exercise habits were formed. These practices included taking up at-home exercise using equipment they had available, swapping from gym visits to walks or runs outside, and for others, seeking out online classes or instructional videos.

If anything, we are doing a bit more exercise at home. We're doing a bit of yoga in the morning and getting out and about. Doing some runs at lunchtime here and there. That's kind of changed for the positive. (James, aged 26)

Using online resources for exercise or fitness training was not always ideal. Several participants noted that they missed the company of other people and the motivation or learning that exercising together could bring. As Natalia (aged 67) observed:

The lack of going to a gym to my class of Zumba three times a week, that is something that I really miss. There's Zumba videos on YouTube [but] well, it's not the same to do things online than interact with people and being in a room, seeing how others are doing, and to learn or copying, or if you lose the steps.

### 5.2. Habituating to preventive measures

When participants were discussing how they had responded to government warnings about COVID prevention, increased hygiene measures such as frequent handwashing after public outings were frequently mentioned. Responding to government warnings about staying at home as much as possible and maintaining distancing from others, several people said that they had made fewer trips to public places (for example, shops) to avoid being around other people in crowded indoor spaces. As a consequence of these warnings, for most people, the home or open outdoor spaces were considered to be place of far greater safety than indoor public spaces. People reported engaging in fewer trips to shopping centers and mainly shopping in their local area or ordering goods online.

During the stage when the virus was more prevalent, I stayed away from shops as much as I could except for essentials. My daughter offered to do my shopping, but I'm a bit independent and prefer to duck in and out of shops rather than spend a lot of time looking around. (Faye, aged 73)

Already in this early phase there were major differences in how people observed others behaving in public: some maintaining highly protective practices and others apparently feeling little sense of fear or risk and therefore readily dropping their “hygienic” behaviors. These comments suggest that people were closely observing others' behaviors and practices and assessing whether a public space felt “safe” to enter. A common response from participants when recounting challenges they had faced in relation to COVID expressed frustration and irritation with other people's behaviors when they were assessed as not engaging in appropriately safe practices. This was particularly the case with social distancing: participants felt that they were doing their best to stand at a safe distance (the recommended 1.5 m) away from other people but that others were not as vigilant and therefore exposing people to infection.

When I have gone out to the supermarket recently, it's like social distancing no longer applies. I've found that really frustrating… I guess I'm a kind of a rule follower, but also, I just know that [the virus] is still there and that you still need to be doing it. People just seem to be over it, they've decided otherwise for themselves, and I'm concerned about that. (Joe, aged 41)

Several people also mentioned the hoarding and panic buying behaviors that received a high level of media coverage during this period.

People were crazily shopping and buying a heap of stuff. You tell yourself, yeah, look, I don't need all that. But then if you're seeing other people do it and there's nothing on the shelves, you actually start to then say, well, I'm going shopping once a week every now and then, I should probably stock up. Because if I go again and there's nothing there that I need then that's going to be an issue. (James, aged 26)

In some cases, participants recounted their worries about accessing essential goods and services: particularly if they were forced to stay in their homes due to mobility difficulties or health conditions. For people with such disabilities or conditions, a frightening feeling about dependency on others was sometimes described. For example, Hannah (aged 47), lives with multiple chronic illnesses. She described the loss of independence she felt during lockdown:

I thought, well, I got told I need to self-isolate, so I couldn't just go down the street and I couldn't just go to the supermarket. Having stuff like click and collect [grocery ordering] was better than nothing, but I was, yeah, trying to hunt down people who could, you know, can you pick this up or could you pick that up?

### 5.3. Social isolation and loneliness

Participants discussed the effects of social isolation during the lockdown, the need to cancel their travel plans and the loss of regular leisure activities such as going to the gym, social clubs or the pub. They reported seeking greater contact through phone and video calls, messaging or social media interactions.

Communicating with family because of the virus has been a positive impact because I'm more likely now to phone them than I was before. We're a bit more fluent with Zoom and WhatsApp–what do you call it, video platforms. That's been positive for me. (Greg, aged 69)

However, several people noted that while they appreciated being able to keep in contact with others using remote methods, this was not as valuable as being able to share the same physical space with others. For these participants, the multisensory embodied engagements and feelings of intimacy they had with other people were greatly diminished in online encounters. Peter (aged 39) told us, for example, that:

I don't like Zoom meetings, they're very impersonal to me. I don't like the screen–it gives me eyestrain. I miss physical touch, the fact that you shake a hand or pat a back, or give somebody a hug when you greet, that definitely took a hit.

These feelings were expressed even by young people, who are popularly assumed to be digitally literate to the point that they prefer online interactions or messaging. Amala (aged 21), described the loss of connection she felt she had with her friends:

I try to catch up with my friends through internet and through video calls but it's different–I lost some friends I'd say. Like the connection we had, I kind of lost it.

For many people, these feelings of isolation and loneliness–the physical separation from others–were described as badly affecting their mental wellbeing. Hannah (aged 47) told us that she was already living with bipolar disorder and struggled with coping during lockdown. However, she also noticed friends and online contacts were feeling down as well:

Well it's isolating–and being in isolation does a pretty good number on your mental health as it is. Not just me. I have contact, like, I've got Facebook contact with various people from various groups and everyone's mental health has declined.

People accustomed to socializing outside the home suddenly found that their options were limited to home-based activities. They were forced to rely mostly on other occupants of their homes or neighbors for in-person contact. Participating in shared family activities, checking in with others, and strengthening ties with neighbors and local community was important for some in ameliorating isolation:

When [my kids] finish schoolwork, we've been able to do more bike riding. And from a physical fitness point of view, and away from devices, this has been definitely positive. (Kevin, aged 44)

Several people, like Riley (aged 29), described spending more in-person time with their immediate neighbors in the attempt to maintain some kind of social contact, and to seek to regain a sense of normality in their lives:

We hang out besides the firepit and have a beer or something. I've talked with my neighbors, tried to make food for my neighbors, they've made food for me. It's been a lot more focus to my particular neighborhood because that's people that you can socialize with without taking a bunch of risks.

### 5.4. Changes to work and education

Many people had lost their paid work or volunteer work due to the enforced closing down of businesses and community organizations. Others had transitioned to working from home and had learned to communicate with their work colleagues using video meetings. Some people found that they were experiencing an increase in their workload, but others had lost some or all of their paid work.

Those in unpaid work such as volunteering in community organizations or family caring often felt a great sense of loss of purpose and identity when they were no longer able to participate in these activities. Tilda (aged 56), for example, described the loss of meaningful roles caring for her grandchildren:

[Caring for my grandchildren] was a big part of my life. […] So that was a huge, huge–yeah, it was a huge, complete change to my life… I'm a really service-oriented person. I really enjoy doing things for other people and I find a lot of self-worth in that, I guess.

Other people expressed worries about finding re-employment after a job loss or reduction in income, with poverty a real threat. Amy (aged 27) had just started working casually in hospitality before lockdown restrictions were established, and she then lost her job. She was ineligible for any government assistance because she does not have permanent resident status in Australia. Amy was struggling with worries about her unemployment status. She feared that she may not get another job and is not sure how she might earn money or pay her rent and power and grocery bills in the future.

Now I'm competing with everybody who lost their job in hospitality. So, I don't have any hope–I mean, I'm still applying for everything I can, but I have absolutely no hope that I'm going to find anything.

Similarly, Tahlia, aged 22, unemployed and with caring responsibilities for two younger siblings, told us about the difficult process she had commenced pre-COVID to access support for her anxiety and depression and trying to find secure paid work. She expressed her frustration that her life (and the hopeful future she envisaged) had been “put on hold” and the sense of being in limbo she felt:

So, I finally took that very long step, and then–yeah… I just feel kind of annoyed, I suppose. I don't know when–things are still not back to normal. We don't know when things are going to go back to normal.

People caring for young children faced the challenges of supervising school from home. Danielle, aged 41, is one example. She described the stress of caring for her 4-year old son and helping her 6-year-old son with his online education when schools closed down, while trying to maintain her paid work as a mental health support worker, also from home. Danielle's relationship with her partner broke down during this time, and she also talked about the stress of being forced to cohabitate with him during the lockdown.

What with working and looking after children–because I mean, my children are delightful, but usually I don't have them around that much, not all the time. Then yeah, there was not enough breaks. Not enough time to be on my own, which I seem to need.

Danielle said that she often experienced a strong urge to leave the house and get outside for a walk or a run, just to be by herself for a while. Caring obligations and the intrusion of work and family into all parts of her life often left very little time and space for self-care. She felt trapped inside.

I'm sort of rammed up against all these people who normally, we have a lot more space with. One of whom I'm not in a relationship with anymore. So, getting out and going for a run was really about getting a break from all that. So, I would do it daily, if I could, but I can't, because at the moment I've got my youngest at home with me.

### 5.5. Little change to life

There were a number of participants who did not experience the pandemic as particularly disruptive, reporting little change in their everyday lives due to COVID. Their accounts indicated a continuation of life “as normal” as they described their daily routines and practices as changing only minimally. For some people, this was because they were already living in relatively socially or geographically isolated situations due to inhabiting a remote location, engaging in the quiet rhythms of retirement. Christine (aged 68), retired and living on a large rural property, is one such participant. She told us that her preference for solitude and a secluded life meant that for her and her partner, daily living was almost unaffected.

We've been practicing for this for years. We used to go out and shop once a week, we now go once a fortnight. That's about the main change. That's it... We've never tended to go anywhere anyway… It really, really hasn't impacted us much at all.

For participants like Greg (aged 69) and also living in a rural region, the outbreak seemed remote. Greg recounted that his everyday life has changed very little since the crisis began, in part because he was already socially isolated, without local contacts. He and his partner had only recently moved into a rural area and had not had an opportunity to meet the local people. He also noted that he is not someone who goes out to pubs or bars to socialize.

I've moved into a small rural village, and so have no friends around me. I don't know anyone. At the start of the virus, I was still pretty socially isolated because of the move. Yes, I was living with my partner, but essentially there's been very little change to my life.

There was another subsection of participants who expressed feelings of invulnerability to being affected by the pandemic. They mentioned factors such as their existing good health that they thought protected them from contracting the virus or becoming seriously ill, leading them to see little sense in changing their habits in response to the threat of COVID infection.

For some of these people, their sense of low risk was based on their understandings that the virus was only affecting the health of “other” people: people living in other countries where there was much greater incidence of COVID (for example, China, Italy and Spain at that period in the pandemic) or those who were already vulnerable because of pre-existing conditions or their age. Some participants thought back to news reporting of seasonal influenza outbreaks and drew parallels with these events. They noted that even though such outbreaks are often serious, they did not feel at risk from influenza, and therefore discounted COVID as a personal threat.

I don't get sick, and this coronavirus… it's just another flu. So I'm pretty well fit in that regard. So I don't really worry about it too much at all. I go about my life as normal. (Dave, aged 54)

### 5.6. Imagining post-COVID life

The final question in the interview asked: What do you think your way of life will be like once the coronavirus crisis has passed? Will it go back to the way it was before–or be different in important ways? For many participants, the experiences of living through the first 6 months of the COVID crisis had provoked reflection on their way of life, their values and those of other Australians and people worldwide: the personal, the national and the global. The participants questioned what “normal life” would mean once the crisis had passed.

For some, especially for younger people and those who had experienced the most disruption to their lives, returning to pre-COVID life was something they hoped for and expected, even if it may take some time. This optimism was expressed by Matthew (aged 23):

[I'm at] uni and everything is going to start back up very quickly and I'm going to jump onto it right as everything is pumping out full pace again. I'm quite excited to see what new industries are coming out and how we sort of move around the world and stuff like that.

Many others were less optimistic, raising concerns about the risks of going “back to normal” too quickly and forgetting to take necessary precautions:

I would hope to think that there would be some lessons learned and that things would be done differently, but my experience walking downtown lately, it's like everyone's just gone back to normal. I'm quite surprised. […] I'm thinking, wow, people are not taking any of the things seriously. (Sarah, aged 54)

These participants hoped that changes in ways of being (for example, social distancing, hygiene practices, and changes in the built environment) would be enduring. In the future, Kim (aged 70) observed, people may be more likely to stay home more and socialize less as they have enjoyed a slower pace of life and being less busy.

I think people have got used to staying home… So I wonder if there'll be less people going out and doing those social things. Even going for coffee, going for lunches and that. I wonder if people have kind of got out of the habit of that and won't return to it.

Some participants also talked about their fears of the longer-term consequences of the crisis for the economy, and the ripple effects for people's financial security, mental health and quality of life.

I think there's still going to be a lot of people out of work, and I think the economy is going to take quite a few years to recover… I do worry about the number of people that will commit self-harm to get out of the problem. And that worries me and so does the mental health issues. (Darren, aged 64)

Participants also reflected more broadly about whether there would be any long-term changes to societal values, including raising more awareness about global issues such as climate change, inequality and individualism.

I think people are going to be more–they're going to start question about the future and hopefully think about more spiritual values than material values. I think people are going to value their community more, instead of taking everybody for granted. (Tom, aged 55)

Some hoped that the camaraderie or solidarity that people had shown to each other during the crisis might persist. This included more focus and attention on issues of social justice and social welfare, including a change in attitudes and treatment toward those who rely on social welfare support, as well as a stronger sense of community. As Max (aged 52), put it:

I don't like the concept of just snapping back to normal, because whatever the old normal was, it wasn't particularly good.

These participants discussed their hopes that valuable lessons would be learnt that would mean that governments and societies were better equipped to deal with similar crises in the future.

The other thing that I hope will change, is that people will be more aware of the fact that this system is not working so well for the wellbeing of the environment and society. (Natalia, aged 67)

## 6. Discussion

The findings from our study build on previously published research by drawing attention to the complexities of how living through the initial months of the COVID crisis affected people living in different locations and socioeconomic circumstances within the same country. As previous studies conducted in countries other than Australia have found, there are specific geographical and other sociodemographic dimensions (2, 5, 9, 10) that structure people's experiences, and indepth qualitative research is able to draw out such complexities. In our research, such factors as people's gender and life stage, their social relationships and obligations as well as their histories of illness made visible some of the unequal social and economic effects of the pandemic. Most of our participants described the shock of having “normality” challenged once COVID restrictions were implemented and warnings about risk were issued by governments and health agencies. What is particularly notable however is that our participants fell into three roughly equal groups: (i) those who found the lockdown and associated restrictions very difficult; (ii) those who reported feeling barely affected by these conditions; and (iii) those who found benefits to the “slowing down” of life during this period. Socio-spatial dimensions such as in which state or territory people resided, whether they lived in metropolitan or rural communities, their age and life stage, whether they were employed and in what occupation, and whether they were living with pre-existing health conditions were associated with how vulnerable people felt to the risk of COVID and how badly affected they were by the national lockdown and its associated socioeconomic impacts.

In the early period of the COVID crisis, people across the world found themselves confronting a multitude of emotional challenges posed by confronting the risks of a novel deadly infectious disease and the restrictions imposed by authorities to limit its spread (6–8). Previous research conducted in Australia during the first COVID wave also showed a disruption of routines and worsening of mental health across the general population (21, 22). Our findings support these international and national studies but further identify that the crisis affected our Australian participants' experience of daily life quite differently depending on their individual circumstances, extending Australian studies that have pointed to the difficulties faced by groups who were already vulnerable and marginalized by virtue of their health status (25) or sexual identity (24). Among our participants, the disruption to everyday life associated with the pandemic contributed to a complex mix of feelings and emotions including worry, fear, anger, frustration, sadness, uncertainty, grief, boredom and loneliness. These feelings were closely entwined with participants' sense of how much at risk they personally felt from infection and their observations of others' behaviors. Some participants' accounts suggested that they felt invulnerable to the crisis because they perceived it to be a distant threat. Many others though, especially those who discussed long-term physical and mental health conditions, expressed a deeply felt sense of vulnerability, fear and anxiety about contracting the virus. This group of participants expressed various worries spanning concerns about access to essential goods and services like groceries and medications, finding re-employment after a job loss or feeling safe in public places.

Similar to previously published international (12) and national (23, 32) research, our findings also identified such sociodemographic factors as gender and responsibility for care affecting people's quality of life during lockdown. The participants who were living in crowded circumstances or precarious housing, where it was difficult to leave their homes to find time alone, or who those were reliant on others for basic needs, reported feeling most affected by the lockdown conditions. Participants who had lost their jobs or income, people living with chronic health conditions and parents dealing with children learning from home reported greater hardships and stresses as they struggled with the ramifications of COVID restrictions and closures. The absence of opportunities for socializing, engaging in meaningful work (paid or unpaid) and caring resulted in some participants losing their sense of meaning or purpose in life. As international studies have identified (11), on the whole, young people in our research were more adversely affected by lockdowns and other disruptions to their lives than were people at the opposite end of the life course.

Our findings further identified changes in people's social connectedness, both in how they interacted (across online/offline spaces) but also in the value that they placed in their relationships. Participants talked about the increasing importance of authentic and meaningful relationships with their friends and family and the emergence of new spaces and forms of belonging (for example, new connections with neighbors). Some people said that the crisis made them feel a sense of comfort and solidarity with others through a shared experience. For others, however, especially those who lost relationships and lost the routines and structures that provided the framework for social life, the COVID crisis and ensuing restrictions on mobilities and the national lockdown together contributed to the most difficult and isolating experience of their lives. Physical distancing measures transformed how participants interacted and felt with one another. The absence of being with others evoked profound feelings of loneliness and isolation for many participants and hopelessness about how long the crisis and lockdown restrictions would continue. Being seen, recognized and acknowledged by others, and valued by them, was of critical importance in affirming a sense of belonging and connectedness. Similar to previous Australian research, we found that people used digital technologies to help with feelings of loneliness (29) or to continue exercise regimens (31), but also identified the limitations of these solutions. Like the middle-aged women in the study by Lunnay et al. (26), several participants reported engaging in greater alcohol consumption to counter the stresses of the pandemic.

Complementing other Australian studies' findings (27, 28, 30, 32) on the “silver linings” of the pandemic experience for Australians, some of our participants also described adapting their routines in ways that helped them deal with the stresses of the first wave and reported benefits of a slower lifestyle during lockdown. Those participants who reported few changes to their lives were older retired people in good health already living a quiet life in a rural area. They said that they had coped well with lockdown conditions and had even found some degree of benefit from the changed circumstances, appreciating nature and a less hectic pace of life. Such individuals did not need to worry about financial pressures, lack of space, or caring for children at home while juggling other demands.

What is particularly novel in our research is the question we asked at the end of the interview about how our participants imagined the future once COVID had passed. Their responses offered further insights into their experiences and feelings about the pandemic in the early stages. People who had experienced COVID-related risks and restrictions as seriously disrupting their daily lives, (those with constrained resources, precarious work, confined living conditions, increased caring responsibilities, the socially isolated) described being stuck in the precarious and uncertain present as distressing and expressed a longing for a return to normal (their pre-COVID lives/past-realities). For them, the crisis disturbed (and for many dismantled) their vision of a hopeful future and set them back in their imagined trajectory toward secure incomes, work and relationships. The future, like the present, now felt much more uncertain or unknowable for many. The future had transformed from a space of anticipated stability to an unknowable and uncertain temporal space (38). Others, in contrast, experienced pandemic life in more neutral or positive ways: in some cases, simply as a continuation of their quiet but contented pre-COVID lives. There was yet another group of participants who saw the crisis as an opportunity for renewal, enrichment, and growth. They desired a “new normal,” articulating their hopes for an optimistic post-pandemic future for themselves and for society more broadly. These participants were more likely to reflect on the positive lessons that the pandemic had given them or society, including the valuing of the social over the material in living well. Notably, however, this viewpoint was expressed from a position of privilege, as it tended to be articulated by those who experienced least social disruption or socioeconomic disadvantage during the crisis.

## 7. Conclusion

Our study's findings draw attention to the importance for public health policy makers and practitioners of recognizing that experiences of COVID restrictions and lockdowns may vary significantly, even within nations. The temporality of the crisis is also important to acknowledge. As the COVID crisis continues, people have had to confront and manage a constantly changing risk and public health policy environment. Public health practices and communication need to acknowledge the dynamic and complex nature of publics' understandings and responses to COVID. This rapid change in Australian society in COVID spread and the subsequent illness, death and disruptions to essential services such as food supplies, travel systems and education provision provides a stark example of the importance of social and public health policies in preventing not only disease and excess deaths but also social and economic disruptions. There is a need for continuing insight into the ways in which the pandemic has been experienced by people across the globe and within individual countries and regions in different ways.

## Data availability statement

The raw data supporting the conclusions of this article will be made available by the authors, without undue reservation.

## Ethics statement

The studies involving human participants were reviewed and approved by UNSW Sydney Human Ethics Research Committee. The patients/participants provided their written informed consent to participate in this study. Written informed consent was obtained from the individual(s) for the publication of any potentially identifiable images or data included in this article.

## Author contributions

DL conceptualized, designed and led the study, analyzed the data, and led the writing of the manuscript. SL conducted the interviews, analyzed the data, and contributed to the writing of the manuscript. All authors contributed to the article and approved the submitted version.

## Supplementary material

Supplementary Material Interview Schedule.
