# Supplementary material for: Australians' experiences of COVID-19 during the early months of the crisis: A qualitative interview study
Source: Front Public Health. 2023 Feb 23;11:1092322. doi: 10.3389/fpubh.2023.1092322 (PMC9995885; doi:10.3389/fpubh.2023.1092322)
Supplement: Supplementary Material — Interview Schedule. [file Data_Sheet_1.docx]

AUSTRALIANS’ EXPERIENCES OF THE COVID-19 CRISIS: A SOCIAL RESEARCH STUDY

**Interview Schedule**

*Sociodemographic questions*

1. Age (in years)
2. What country were you born in?
3. If born in a country other than Australia – how long have you been living in Australia?
4. What part of Australia do you currently live in (major city/regional city/rural/remote area)?
5. What do you do for a job (pre-COVID and now)?
6. What is your highest level of education (Year 10 or below, Year 12, technical certificate, some university, completed university degree)?
7. What is your ethnicity/racial background (white Australian, Aboriginal/Torres Strait Islander, other self-described …)?
8. Do you currently have a long-term health condition or disability? (please specify)
9. What are your current living arrangements? (living alone, with family members [please specify who they are – e.g. partner, children under 18, adult children, parent, grandparent], with housemates [how many?])

*Questions about COVID-19*

1. Think back to when you first heard of coronavirus – when and how did you first hear about it? How did you feel about it at that time? (Probes – worried, not interested, unconcerned, didn’t think it was relevant to me … any other?)
2. Since that first time of hearing about coronavirus – what has been the most helpful or useful source of information for you to learn about the virus? (Probes: news reports, word-of-mouth from friends or family, your doctor, social media, government-provided information – any other?)
3. What has made these sources so helpful or useful for you? Please explain.
4. How has your everyday life changed due to the coronavirus? Please explain. (Probes – social relationships, leisure and exercise pursuits, sleep patterns, children or partner at home all day, socialising … any other?)
5. Have you or anyone you know been infected with the coronavirus? ***If yes*** – please explain who it is (Probes: partner, family member, friend, someone from work, any other)? How has your life changed because of this infection?
6. Have you lost your job due to the coronavirus? ***If yes***, please explain***. If no***, has your job changed in any way? ***If yes***, please explain.
7. What, for you, have been the hardest or most challenging aspects of dealing with life during the coronavirus? Please explain.
8. What has helped you deal with these hard or challenging aspects? (Prompts: spending time with family members at home, distractions such as cooking, reading, watching TV, playing games, going for walks, mediation, indoor exercise, telephoning friends or family, using social media or video chat services … any others?)
9. Please explain how these have helped.
10. Have you used any government or non-government services to help you in the coronavirus crisis? ***If yes*** – please explain what they are and how they have helped you.
11. What do you think of the ways the Australian and state governments have handled the coronavirus crisis? What have they done well? What could they have done better?
12. What do you think your way of life will be like once the coronavirus crisis has passed? Will it go back to the way it was before – or be different in important ways?
13. That is the end of our questions. Do you have any other comments you’d like to make about how the coronavirus crisis has affected you?
